# Single Anastomosis Duodenoileal Bypass or Roux-en-Y Gastric Bypass After Failed Sleeve Gastrectomy: Medium-Term Outcomes

**DOI:** 10.1007/s11695-021-05609-1

**Published:** 2021-08-16

**Authors:** Phillip J. Dijkhorst, May Al Nawas, Laura Heusschen, Eric J. Hazebroek, Dingeman J. Swank, René M.J. Wiezer, Edo O. Aarts

**Affiliations:** 1 Dutch Obesity Clinic, Huis ter Heide, The Netherlands; 2 Department of Surgery, St. Antonius Hospital, Nieuwegein, The Netherlands; 3 Department of Surgery, Rijnstate Hospital/Vitalys Clinics, Arnhem, The Netherlands; 4 Department of Surgery, Haaglanden Medical Center/NOK-West, The Hague, The Netherlands; 5 WeightWorks Clinics, Amersfoort, The Netherlands

**Keywords:** Sleeve gastrectomy, Single anastomosis duodenoileal bypass, SADI, Roux-en-Y gastric bypass, RYGB, Weight loss, Quality of life, Complications, Micronutrient deficiencies

## Abstract

**Background:**

Although the sleeve gastrectomy (SG) has good short-term results, it comes with a significant number of patients requiring revisional surgery because of insufficient weight loss or functional complications.

**Objective:**

To investigate the effectiveness of the single anastomosis duodenoileal bypass (SADI-S) versus the Roux-en-Y gastric bypass (RYGB) on health outcomes in (morbidly) obese patients who had previously undergone SG, with up to 5 years of follow-up.

**Methods:**

Data from patients who underwent revisional SADI-S or RYGB after SG were retrospectively compared on indication of surgery, weight loss, quality of life, micronutrient deficiencies, and complications.

**Results:**

From 2007 to 2017, 141 patients received revisional laparoscopic surgery after SG in three specialized Dutch bariatric hospitals (SADI-S *n*=63, RYGB *n*=78). Percentage total weight loss following revisional surgery at 1, 2, 3, 4, and 5 years was 22%, 24%, 22%, 18%, and 15% for SADI-S and 10%, 9%, 7%, 8%, and 2% for RYGB (*P*<.05 for 1–4 years). Patients who underwent RYGB surgery for functional complications experienced no persistent symptoms of GERD or dysphagia in 88% of cases. No statistical difference was found in longitudinal analysis of change in quality of life scores or cross-sectional analysis of complication rates and micronutrient deficiencies.

**Conclusion:**

Conversion of SG to SADI-S leads to significantly more total weight loss compared to RYGB surgery with no difference in quality of life scores, complication rates, or micronutrient deficiencies. When GERD in sleeve patients has to be resolved, RYGB provides adequate outcomes.

**Graphical abstract:**

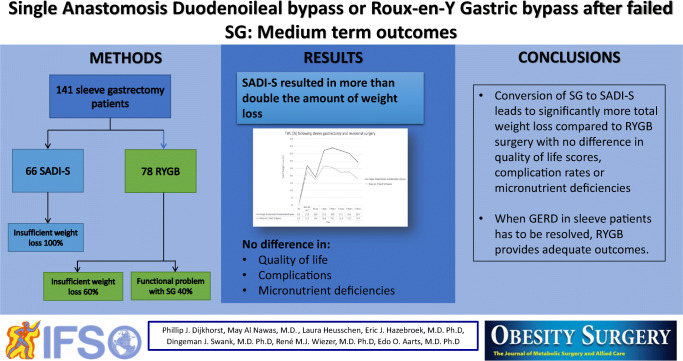

## Introduction

The sleeve gastrectomy (SG) is the most frequently performed bariatric procedure to treat (morbid) obesity, accounting for 55.4% of all bariatric procedures worldwide according to the latest IFSO global registry report of 2018 [1]. This comes as no surprise as the SG is a relatively easy to perform surgical procedure when for example compared to the Roux-en-Y gastric bypass (RYGB), with nearly as good results for weight loss and slightly lower complication rates [2]. Despite of its positive results in the first years following surgery, long-term data show revisional surgery rates of up to 22.6% [3–5]. The two main reasons for revisional surgery after SG are insufficient weight loss and gastro-esophageal reflux disease (GERD), of which the first one is most common.

Although many surgical options are available for patients who require revisional surgery after SG, the optimal procedure remains elusive. In cases where GERD is the main complaint and additional weight loss is not the primary goal, it is generally advised to perform a RYGB as this has proven to be the most effective treatment for the resolution of GERD symptoms [6–8]. This effect can be explained by surgical “removal” of the pylorus from the equation, resulting in a lower pressure system and thereby promoting gastric (pouch) emptying. When insufficient weight loss is the main reason to choose for revisional surgery, adding additional restriction can be achieved with a revisional sleeve gastrectomy (Re-SG). One might also look at more malabsorptive procedures. These include the RYGB, one anastomosis gastric bypass (OAGB), and single anastomosis duodenoileal bypass (SADI-S). However, it can only be speculated which surgical option is best. Besides, there is no “one fits all” approach when it comes to revisional surgery. In choosing the most suitable surgical option, it is always the goal to create an optimal equilibrium between weight loss, complications, and micronutrient deficiencies, as well as taking quality of life after surgery into account [9, 10].

As there is little data published on the outcomes of revisional surgery after the SG, often with small sample sizes, there is a large need for data with larger sample sizes and longer follow-up periods. Previously, the short-term outcomes (0–2 years) of the RYGB and SADI-S after SG in the similar set of patients have been reported [11]. We found that SADI-S after SG led to significantly more weight loss, while complication rates and micronutrient deficiencies were similar for SADI-S and RYGB. The aim of the current study is to provide medium-term results of the SADI-S versus RYGB as revisional surgery after the SG by comparing weight loss and other health outcomes (quality of life (QOL), micronutrient deficiencies, and complications) for all patients with 3 to 5 years of follow-up.

## Methods

### Study Cohort and Data Collection

All patients who underwent revisional bariatric surgery for SADI-S or RYGB after SG from 2007 to 2017 were included in this study. The participating hospitals included Center 1: Rijnstate hospital in Arnhem, Center 2: St. Antonius hospital in Nieuwegeinand Center 3: Haaglanden Medical Center in The Hague,all located in The Netherlands. The national Medical Ethics Review Committee and the local review committee of the individual hospitals approved the protocol of this study. Data were collected retrospectively from medical records and supplemented by data from the standardized lifestyle program that is provided by the (blinded) regarding weight loss, laboratory results, and questionnaires. After data collection at the three individual locations, all data was anonymized.

Prior to any bariatric procedure, patients are extensively screened by a multidisciplinary team to check whether they are eligible for surgery. Before considering a SG, it is especially important for the surgeon to ask for symptoms of GERD and to analyze the pre-operative questionnaire that every patient fills in on GERD symptoms [12]. When patients have complaints of GERD, they are strongly advised to choose for a RYGB instead of a SG. After SG, the decision to choose for SADI-S or RYGB as revisional surgery was made in a shared decision-making approach with the surgeon and patient. If a patient presented with GERD or an anatomical problem after SG, such as a stenosis, a RYGB would be strongly advised. In all other cases, personal preferences of the surgeon and patient led to the decision for either SADI-S or RYGB. The operational techniques and post-operative management have been previously described [11]. From 2014 to 2017, all SADI-S operations were performed with a common channel length of 250cm. However, in 2017 two of the participating centers changed the common channel measurement to 300 cm, leading to four SADI-S patients with a common channel length of 300cm.

Inclusion criteria consisted of age 18–65 years, prior SG surgery, BMI >35kg/m^2^, and all other criteria described in the European guidelines for bariatric surgery by Fried et al [13]. Patients who experienced complications from the SG could have a BMI <35kg/m^2^. Exclusion criteria were known unstable malignancies or chemotherapy and less than 3 years of follow-up. For weight loss results, patients who were operated without insufficient weight loss as a main indication for surgery (such as conversion of SG to RYGB because of a functional problem) and pregnant women (from the time of pregnancy until 3 months after parturition) were excluded from the analyses.

### Outcome Definition

The primary outcome of interest was weight loss following revisional surgery, defined as percentage total body weight loss (%TWL, weight loss in kilograms at a follow-up time point divided by weight in kilograms measured before revisional surgery). Pre-operative weight was measured on the day of surgery. Follow-up weight was measured at 1.5, 3, 6, 12, 18, and 24 months following revisional surgery and yearly thereafter.

Secondary outcomes were resolution of GERD, complications, micronutrient deficiencies, and quality of life. Resolution of GERD was defined as no medical record of complaints and/or no medication such as proton pomp inhibitors (PPIs) at the last follow-up moment. Medical records were traced from the time of surgery until 5 years of follow-up to search for complications that were related to the bariatric surgical intervention. Laboratory results were collected 6 and 12 months and then yearly after revisional surgery to be analyzed for deficiencies. Patients were included when data on a specific nutrient was available for at least one follow-up moment. The guidelines of the American Society for Metabolic & Bariatric Surgery for the surgical weight loss patient, updated version on micronutrients, were used as a reference for normal and deficient micronutrient lab ranges [14]. When lab ranges for micronutrients were not covered in this guideline, the reference value from the Dutch Association for Clinical Chemistry and Laboratory Medicine was used [15].

QOL was assessed in two out of three centers by using the SF-36 questionnaire on self-rated physical and mental health starting in 2011 [16]. This questionnaire consists of 36 questions and nine scales from which two subtotals could be calculated: physical health summary (PHS) and mental health summary (MHS) score. Besides, health change over the previous year is one out of nine subscales in the questionnaire. A score of 50 is neutral, above 50 is a positive change, and below 50 is a negative change. This questionnaire has been validated for patients with obesity [17].

### Statistical Analysis

Retrospectively collected data were analyzed using IBM SPSS version 24 for Windows. Normally distributed values are presented as mean (± standard deviation) and non-normally distributed as median (range). Descriptive statistics were used for demographic variables. Weight loss was only analyzed for patients in which insufficient weight loss was the primary indication for surgery. Differences between groups in weight loss were analyzed by Student’s *t* test for cross-sectional data and mixed models for longitudinal data. To control for potential confounding, the following variables were added to the longitudinal model: gender, age at revisional surgery, pre-operative weight before revisional surgery, lowest weight after SG and before revisional surgery, and time interval between SG and before revisional surgery. Differences between groups in the occurrence of micronutrient deficiencies and complications were analyzed using the chi-square test. The SF-36 scores for quality of life were analyzed using a Student’s *t* test for cross-sectional data and mixed models for longitudinal data. A *P*-value of <0.05 was considered statistically significant.

## Results

From 2007 through 2017, 141 patients underwent revisional surgery for SADI-S or RYGB (center 1, *n*=54; center 2, *n*=39; center 3, *n*=48). SG was converted to a SADI-S in 63 patients and to a RYGB in 78 patients. Indications for a RYGB were insufficient weight loss in 39 patients (50.0%), functional problems related to the SG in 32 patients (41.0%), or a combination of both in 7 patients (9.0%). All SADI-S operations were performed after 2014 as then this procedure was introduced in the Netherlands. This is in contrast to the RYGB which was performed scattered between 2007 and 2017. An overview of baseline characteristics is given in Table 1.

**Table 1:** Baseline characteristics

	SG		SADI-S		RYGB^1^	
	*N* = 141	± SD or range	*N* = 63	± SD or range	*N* = 78	± SD or range
Age (years)	41.9	±11.1	43.6	±10.6	46.0	±11.1
Percentage female	80.9%		84.1%		78.2%	
Weight, kg	152.6	±30.7	129.3	±21.5	112.3	±25.5
BMI, kg/m^2^	53.1	±9.7	44.9	±6.2	39.1	±8.0
Operative time, minutes	70.0	27–155	83.5	38–199	75.5	39 - 212
Hospital stay, days			1	1–8	2	1–25
Years until revision			3.2	1.0–11.9	2.0	0.3–6.8
Follow-up after revision, years			4.6	3.2–5.6	7.8	3.2–13.8
Comorbidities (%)						
Hypertension			48.4%		53.9%	
Diabetes Mellitus			21.0%		27.7%	
Dyslipidemia			8.1%		20.5%	
OSA			24.2%		12.9%	
Physical health summary score^2^	47.7	5.0–86.9	77.5	16.3–96.3	60	8.9–92.5
Mental health summary score^2^	66.5	10.1–94.5	73.4	28.5–97.8	64	15.0–93.0

*N* number of patients, *± SD* standard deviation, *SG* sleeve gastrectomy, *SADI-S* single anastomosis duodenoileal bypass, *RYGB* Roux-en-Y gastric bypass, *BMI* body mass index, *OSA* obstructive sleep apnea

^1^RYGB group contains both indications for surgery (weight loss and functional problems) leading to lower average weight/BMI and quality of life scores

^2^Including all patients from which baseline quality of life scores were available

### Primary Outcome: Weight Loss

**Fig. 1:**
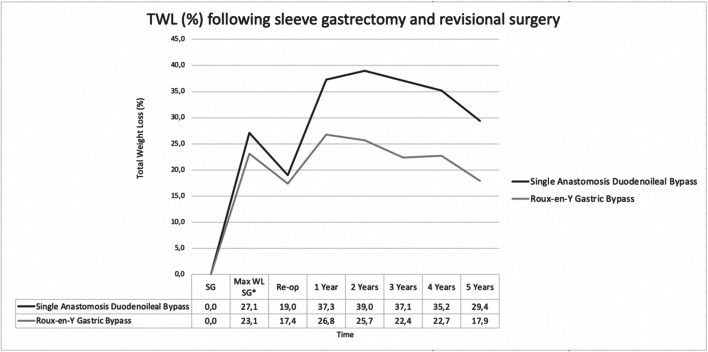
Percentage total weight loss (%TWL) after sleeve gastrectomy and revisional surgery; SG sleeve gastrectomy. Asterisk indicates maximum %TWL obtained after SG and before revisional surgery

Sixty-three SADI-S and 46 RYGB patients were operated with insufficient weight loss as a main indication for surgery. Weight loss defined as %TWL was significantly better after SADI-S at years 1 to 4 when compared to the RYGB (Table 2). Longitudinal data analysis with mixed models revealed an average %TWL over time of 19.8% after SADI-S and 8.1% after RYGB (*P*<.001). Figure 1 shows cross-sectional data on %TWL from the time of SG.

**Table 2:** Percentage total weight loss (%TWL) after revisional surgery

Years post-op	SADI-S*	*N*	RYGB*	*N*	*P*-value
1	22.2 ±9.1	57 (90%)	9.7 ±9.5	36 (78%)	<.001
2	24.4 ±10.4	44 (70%)	9.0 ±11.5	32 (70%)	<.001
3	21.8 ±11.7	36 (57%)	7.2 ±12.5	25 (54%)	<.001
4	17.9 ±12.2	16 (35%)	8.2 ±15.1	21 (48%)	.042
5	15.0 ±22.8	9 (47%)	2.1 ±13.2	16 (40%)	.057

*N* number of patients available for analysis and follow-up percentage, *SADI-S* single anastomosis duodenoileal bypass, *RYGB* Roux-en-Y gastric bypass, *%TWL* percentage total weight loss after revisional surgery, *± SD* standard deviation

*Six SADI-S and one RYGB patient had part of their weight loss data excluded because of pregnancy

To ensure internal validity, the following variables were analyzed as potential confounders for the relationship between %TWL and type of surgery: age at revisional surgery, gender, pre-operative weight before revisional surgery, lowest weight obtained after SG and before revisional surgery, and time interval between SG and revisional surgery. None of these variables was considered to be confounders as they did not change the regression coefficient of %TWL over time in a longitudinal data analysis with more than 10%.

### Secondary Outcomes: Quality of Life, Micronutrient Deficiencies, Complications, and Functional Problems

The number of patients who had filled in the quality of life questionnaire both before revisional surgery and at least at one time point post-operatively was 32 (68%) for SADI-S and 28 (47%) for RYGB. In this group of patients, the pre-operative average physical health summary (PHS) score for SADI-S and RYGB was 67.5 and 56.4, respectively. Longitudinal data analyses revealed that SADI-S patients had an average post-operative PHS score over time of 69.0 versus 67.4 for RYGB patients (*P*=.68). The pre-operative average mental health summary (MHS) score for SADI-S and RYGB was 71.6 and 62.6, respectively. Longitudinal data analyses revealed that the average post-operative MHS score over time was 65.3 for SADI-S and 70.5 for RYGB (*P*=.183). The self-rated score on health change over the previous year at 1 year post surgery was 81.5 for SADI-S and 80.9 for RYGB patients (*P*=.88).

Next, a subgroup analysis of RYGB patients who were operated for functional problems versus RYGB patients who were operated for insufficient weight loss was performed. For the functional problems group, the pre-operative PHS and MHS scores were 51.3 and 60.4. The average post-operative scores over time were 62.2 and 67.8, respectively. For the insufficient weight loss group, the pre-operative PHS and MHS scores were 61.0 and 64.6. The average post-operative scores over time were 73.3 and 72.9, respectively. The difference in post-operative PHS and MHS scores between these groups was not statistically significant.

An overview of the micronutrient serum values is given in Table 3. The table only displays newly formed deficiencies after revisional bariatric surgery. If a micronutrient for a single patient was abnormal prior to revisional surgery, then the specific micronutrient for that patient was not analyzed. In total, 46/60 (76.7%) patients developed a new deficiency in a single or multiple micronutrient(s) after SADI-S and 49/68 (72.1%) after RYGB. More vitamin B12 deficiencies were found after RYGB, while more folic acid deficiencies were found after SADI-S (*P*<.05).

**Table 3:** Pre- and post-operative micronutrient values and deficiencies after revisional SADI-S and RYGB

		SADI-S			RYGB			
	Critical range	Pre-op deficient	Post-op deficient	Post-op (*N*)	Pre-op deficient	Post-op deficient	Post-op (*N*)	*P*-value
Hemoglobin (mmol/l)	M: <8.5 F: <7.5^a^	7 (11.3%)	24 (38.7%)	62	12 (17.9%)	17 (23.9%)	71	.099
Calcium* (mmol/l)	<2.15^b^	2 (5.9%)	14 (23.3%)	60	2 (6.5%)	9 (13.0%)	69	.123
Phosphorus (mmol/l)	<0.9^a^	2 (16.7%)	3 (14.3%)	21	2 (15.4%)	1 (4.8%)	21	.290
Iron (μmol/l)	<8.95^b^	2 (13.3%)	9 (30.0%)	30	4 (20.0%)	6 (17.1%)	35	.260
Ferritin (μg/l)	<20^b^	6 (12.2%)	16 (27.1%)	59	6 (16.2%)	22 (33.3%)	66	.467
Albumin (g/l)	<35^a^	0 (0%)	10 (17.5%)	57	1 (3.9%)	6 (9.4%)	64	.197
Total protein (g/l)	<60^a^	0 (0%)	0 (0%)	21	0 (0%)	0 (0%)	8	NA
Vitamin A (μmol/l)	<0.7^b^	0 (0%)	0 (0%)	30	0 (0%)	0 (0%)	4	NA
Vitamin B1 (nmol/l)	<70^b^	0 (0%)	0 (0%)	46	1 (6.7%)	0 (0%)	36	NA
Vitamin B6 (nmol/l)	<35^a^	0 (0%)	0 (0%)	48	0 (0%)	0 (0%)	35	NA
Folic acid (nmol/l)	<5^a^	1 (2.0%)	6 (10.3%)	58	2 (5.4%)	0 (0%)	66	.008
Vitamin B12 (pmol/l)	<200^b^	8 (16.0%)	2 (3.3%)	60	10 (27.0%)	20 (30.8%)	65	.000
Vitamin D (nmol/l)	<50^b^	15 (30.0%)	15 (24.2%)	62	13 (32.5%)	19 (27.9%)	68	.779
Magnesium (mmol/l)	<0.7^a^	0 (0%)	1 (3.5%)	29	0 (0%)	0 (0%)	15	.467
Zinc	<9.2^b^	0 (0%)	15 (60.0%)	25	0 (0%)	1 (16.7%)	6	.056
Parathyroid hormone	<1.3^a^	4 (8.0%)	1 (1.7%)	60	1 (2.6%)	1 (1.5%)	66	.915

*N* number of patients, *SADI-S* single anastomosis duodenoileal bypass, *RYGB* Roux-en-Y gastric bypass, *M* male, *F* female

^a^Dutch Association for Clinical Chemistry and Laboratory Medicine [14]

^b^Nutritional guidelines for the Surgical Weight Loss Patient 2016 [14]

*Calcium was corrected for albumin if albumin was also determined (Ca_corr_ = total calcium – (0.025*albumin) + 1

Table 4 displays short- (<30 days) and long-term (>30 days–5 years) complications. There was no statistical difference in overall short- or long-term complication rate between SADI-S and RYGB after SG. All surgical procedures were started laparoscopically; however, five had to be converted to an open laparotomy because of technical difficulties such as adhesions, of which two SADI-S and three RYGB procedures. Slightly more RYGB patients reached 5-year follow-up. No peri-operative deaths were observed.

**Table 4:** Short-term and long-term complications

	SADI-S *N* = 63 (%)	RYGB *N* = 78 (%)	Total *N* = 141 (%)	*P*-value
Short-term complications (<30 days)	5 (7.9%)	7 (9.0%)	12 (8.2%)	.826
Readmission	4	5	9	
Abdominal pain/fever	2	3		
Deep vein thrombosis	1			
Wound infection		1		
Persistent nausea		1		
Hematemesis	1			
Reoperation	1	2	3	
Abscess	1			
Anastomic leakage		1		
Other^1^		1		
Long-term complications (>30 days–5 years)	10 (15.9%)	21 (26.9%)	31 (22.0%)	.115
Readmission	2	3	5	
Stenosis^2^		4	4	
Reoperation	8	14	22	
Internal herniation	2	7		
Cicatricial herniation	1	2		
Revisional surgery	3	1		
Re-sleeve	2			
Anastomic leakage^3^	1			
Other^1^		4		
Mortality	0	0	0	

*N* number of patients, *SADI-S* single anastomosis duodenoileal bypass, *RYGB* Roux-en-Y gastric bypass

^1^Other: most common diagnostic laparoscopy for suspicion of internal herniation

^2^Stenosis successfully treated with balloon dilatation in three cases and surgically in one case

^3^Anastomic leakage after re-sleeve operation

Thirty-nine out of 78 RYGB (50%) patients were operated because of a functional problem related to the SG. Indications for revision were GERD in eleven patients (28%) (in which seven suffered from diaphragmatic herniation), dysphagia in 21 patients (54%) (caused by a stenosis/torsion in nine and concomitant with GERD in one), persistent nausea/vomiting in six patients (15%), and fistula in one patient (3%). When a diaphragmatic hernia was present, it was surgically corrected during RYGB surgery and relieved GERD symptoms after surgery in all patients. At the latest follow-up moment, two out of twelve patients who suffered from GERD still had complaints and one still used a proton pump inhibitor. Revision for nausea/vomiting or a fistula resolved symptoms for all patients. Dysphagia symptoms resolved in nineteen out of 21 cases. One patient still had dysphagia in the years following revision and received enteral tube feeding for several months, while another patient had persistent complaints of abdominal pain in combination with dysphagia and was readmitted to the hospital several times even at 5 years post revisional surgery. For both patients, no anatomical abnormalities related to the RYGB were found that could explain their complaints.

## Discussion

To date, there is no consensus on which surgical procedure should be performed after failure of a SG. Therefore, our goal was to compare SADI-S with RYGB following SG on weight loss and quality of life with up to 5 years of follow-up. The present study revealed that SADI-S is more effective in achieving weight loss after a failed SG when compared to RYGB with comparable outcomes in post-operative micronutrient deficiencies, complications, and QOL scores. SADI-S resulted in more than double the amount of weight loss at the latest follow-up following revisional surgery (%TWL after revisional surgery at 1, 2, 3, 4, and 5 years of 22%, 24%, 22%, 18%, and 15% for SADI-S and 10%, 9%, 7%, 8%, and 2% for RYGB). In addition, 78% of patients who received a RYGB never reached an excess weight loss over 50%. However, contrary to our belief after analyzing the results of our previous paper, revisional SADI-S patients do not stay above 20% weight loss on average after 3 years of follow-up.

Few studies have reported on weight loss outcomes of SADI-S as a secondary procedure. Reports on %TWL during the first 2 years after revisional SADI-S surgery vary from 20 to 26% TWL [18–20]. Three studies have demonstrated %TWL results measured from baseline weight at the time of SG and found 38–46% weight loss after SG and SADI-S during 1 to 5 years of follow-up [21–23]. To date, only Sánchez-Pernaute et al (2020) have provided data on weight loss results more than 3 years after SADI-S and reported on the absence of weight regain in this period. Admittedly, this cannot be seen in our data as %TWL drops from more than 20% during the first 3 years to 15% after 5 years. This result may be explained by the fact that only nine patients were included at the 5-year follow-up moment. The poor weight loss results after conversion of a SG to RYGB are in line with those reported in literature. Studies that recommend performing a RYGB after SG for insufficient weight loss usually only report on short-term outcomes [24]. The few long-term reports on %EWL do not often exceed more than 50%, and BMIs under 40kg/m^2^ are seldomly seen [25]. In our opinion, weight loss results after RYGB are far worse than those after SADI-S. RYGB should therefore not be the recommended procedure after a failed SG due to weight regain or insufficient weight loss.

To the best of our knowledge, quality of life after revisional SADI-S or RYGB surgery has not been studied before. The (blinded) has implemented quality of life follow-up for bariatric patients since 2011. Unfortunately, one out of three centers used a different questionnaire than the SF-36, and some of the patients who received a RYGB fell out of the time frame because they were operated before 2011. This leads to an incomplete dataset, and the results should therefore be interpreted with caution. Surprisingly, the QOL scores at baseline were lower for the RYGB group, even when dividing this group into patients with functional problems versus patients with insufficient weight loss. It is possible that patients with higher QOL and weight at baseline were more likely to make the decision for SADI-S which can be seen as a more invasive procedure than RYGB. Both for SADI-S and RYGB, a positive change in health score of 81 and 82, respectively, was found 1 year after surgery (50 is neutral). Longitudinal data analysis of change in mental and physical health score following revisional surgery showed no significant difference between SADI-S and RYGB. It might be speculated that a trend towards better QOL scores is seen after RYGB; however, the groups are too small to draw conclusions from. This trend cannot be explained by inclusion of patients who were operated because of a functional problem with the SG in the RYGB group since a subgroup analysis showed the same improvement in QOL for those operated for insufficient weight loss. We hypothesized that QOL would be better after SADI-S if weight loss is greater with a similar rate of deficiencies and complications. Unfortunately, this cannot be seen in our data.

A concern from procedures with possible significant malabsorptive properties such as SADI-S is the risk of micronutrient and even macronutrient deficiencies. Apparent from our data for both SADI-S and RYGB is the occurrence of a ferritin deficiency and anemia after surgery in about 25–40%, a vitamin D deficiency in 25%, and up to 18% of patients developing an albumin deficiency. Vitamin B12 was significantly more often deficient after RYGB, whereas SADI-S patients developed more folic acid deficiencies. Factors underlying a vitamin B12 deficiency after conversion of SG to RYGB are probably diminished contact of food with gastric acid and decreased secretion of intrinsic factor, unlike after SADI-S where the gastric antrum is preserved [26–28].

The main reason for introducing the SADI-S was to find a better alternative than the original BPD-DS in terms of operative risk and complications. In the present study, no statistical difference was found in overall complication rates after SADI-S or RYGB, as was expected after our previous paper. Internal herniation was found more often after RYGB, as was expected as a RYGB has two potential herniation sites. This is an important outcome, while although the difference is not statistically significant, it is probably due to a type two error and would become significant when more patients would have been available. During the first 5 years after revision, the combined risk for a complication related to the bariatric procedure was 30% of which 58% required surgery. Prior studies have noted that complication rates are generally reported to be higher after revisional surgery compared to primary procedures [29–31]. However, papers do not typically report on re-operation and long-term complication rates, especially after revisional surgery which makes it hard to compare these results. Nonetheless, our data supports the hypothesis that complications are not more common after revisional SADI-S than after RYGB surgery.

Failure of the SG because of a functional problem such as GERD, dysphagia, persistent nausea and vomiting, or a fistula occurred in 39 out of 141 patients (28%). GERD can be seen after a complex interaction of problems including the prevalence of hiatus hernia, increased intra-abdominal pressure, and a malfunctioning lower esophageal sphincter causing decreased gastric emptying which all can be caused by obesity alone [32]. The high-pressure environment created by a SG can cause GERD symptoms to worsen or de novo GERD to occur. A recent study by Mandeville et al. (2017) showed that in one hundred patients, the chance of developing de novo GERD after a SG was up to 48% [33]. By performing a RYGB after a SG, the pylorus is bypassed promoting gastric emptying and relieving pressure which in theory should resolve most of the GERD symptoms, as has also been supported by literature [8, 34–36]. This is also shown in our results as out of 39 patients who were operated because of a functional problem, all but two were free of GERD symptoms. The fact that two patients still had complaints of GERD addresses the complexity of the problem. Converting SG to SADI-S in patients that do not have GERD complaints does not provoke GERD symptoms as the anatomy of the stomach is left untouched during this procedure.

Finally, a number of potential limitations must be considered. As with any retrospective study, there is a risk that missed appointments and insufficient data would be available to answer a research question. Another limitation is the small sample size of SADI-S patients who have reached 5 years of follow-up and the low percentage of data available at this time point. Also, data on QOL was not available for all participating centers and was missing for RYGB patients who were operated before 2011. Accordingly, data that was available for analysis with percentages was given for every outcome to optimize transparency.

Future research should focus on long-term follow-up and emphasize on the role of quality of life. What factors impact quality of life after revisional surgery is a topic that was beyond the scope of the current study but is an important question for future studies. Unfortunately, because of the small sample size that comes along with a very specific group of patients requiring revisional surgery after a SG and their non-compliance to follow-up leading to missing data, it is not possible to draw hard clinical recommendations out of one study. Nonetheless, we can still state that this is the largest scientific comparison of revisional SADI-S and RYGB after SG and it provides a framework for future research on quality of life.

## Conclusion

Converting a SG to SADI-S results in significantly more weight loss than conversion to RYGB up to 4 years post-revision. The average quality of life scores over time did not differ between SADI-S and RYGB, and the rate/percentage of complications and micronutrient deficiencies was similar. Therefore, conversion from a SG into a RYGB is not the preferred procedure unless GERD or functional problems related to the SG are the primary indication for revisional surgery.

## References

[1] Angrisani L, Santonicola A, Iovino P, Ramos A, Shikora S, Kow L. Bariatric Surgery Survey 2018: Similarities and Disparities Among the 5 IFSO Chapters. Obes Surg. 2021;31(5):1937–48. 10.1007/s11695-020-05207-7. 10.1007/s11695-020-05207-7 PMC7800839 33432483

[2] Yang P, Chen B, Xiang S, Lin XF, Luo F, Li W (2019). Long-term outcomes of laparoscopic sleeve gastrectomy versus Roux-en-Y gastric bypass for morbid obesity: results from a meta-analysis of randomized controlled trials. Surg Obes Relat Dis..

[3] Clapp B, Wynn M, Martyn C, Foster C, O'Dell M, Tyroch A (2018). Long term (7 or more years) outcomes of the sleeve gastrectomy: a meta-analysis. Surg Obes Relat Dis..

[4] Guan B, Chong TH, Peng J, Chen Y, Wang C, Yang J (2019). Mid-long-term revisional surgery after sleeve gastrectomy: a systematic review and meta-analysis. Obes Surg..

[5] Neagoe R, Muresan M, Timofte D, Darie R, Razvan I, Voidazan S, Muresan S, Sala D (2019). Long-term outcomes of laparoscopic sleeve gastrectomy - a single-center prospective observational study. Wideochir Inne Tech Maloinwazyjne..

[6] Duke MC, Farrell TM (2017). Surgery for gastroesophageal reflux disease in the morbidly obese patient. J Laparoendosc Adv Surg Tech A..

[7] Gorodner V, Viscido G, Signorini F, Obeide L, Moser F (2018). Gastroesophageal reflux disease and morbid obesity: evaluation and treatment. Updates Surg..

[8] Gu L, Chen B, Du N, Fu R, Huang X, Mao F (2019). Relationship between bariatric surgery and gastroesophageal reflux disease: a systematic review and meta-analysis. Obes Surg..

[9] Mazer LM, Azagury DE, Morton JM (2017). Quality of life after bariatric surgery. Curr Obes Rep..

[10] Raaijmakers LC, Pouwels S, Thomassen SE, Nienhuijs SW (2017). Quality of life and bariatric surgery: a systematic review of short- and long-term results and comparison with community norms. Eur J Clin Nutr..

[11] Dijkhorst PJ, Boerboom AB, Janssen IMC, Swank DJ, Wiezer RMJ, Hazebroek EJ, Berends FJ, Aarts EO (2018). Failed sleeve gastrectomy: single anastomosis duodenoileal bypass or Roux-en-Y gastric bypass? A multicenter cohort study. Obes Surg..

[12] Jones R, Junghard O, Dent J, Vakil N, Halling K, Wernersson B (2009). Development of the GerdQ, a tool for the diagnosis and management of gastro-oesophageal reflux disease in primary care. Aliment Pharmacol Ther..

[13] Fried M, Yumuk V, Oppert JM, Scopinaro N, Torres A, Weiner R (2014). Interdisciplinary European guidelines on metabolic and bariatric surgery. Obes Surg..

[14] Parrott J, Frank L, Rabena R, Craggs-Dino L, Isom KA, Greiman L (2017). American Society for Metabolic and Bariatric Surgery integrated health nutritional guidelines for the surgical weight loss patient 2016 update: micronutrients. Surg Obes Relat Dis..

[15] Laboratoriumgeneeskunde NVvKCe. https://wwwnvkcnl/algemeen-overzicht-referentiewaarden. 2020

[16] Ware JE (1999). SF-36 Health Survey. The use of psychological testing for treatment planning and outcomes assessment.

[17] Karlsen TI, Tveitå EK, Natvig GK, Tonstad S, Hjelmesæth J. Validity of the SF-36 in patients with morbid obesity. Obes Facts. 2011. 10.1159/000333406 PMC6444791 22166753

[18] Bashah M, Aleter A, Baazaoui J, El-Menyar A, Torres A, Salama A (2020). Single anastomosis duodeno-ileostomy (SADI-S) versus one anastomosis gastric bypass (OAGB-MGB) as revisional procedures for patients with weight recidivism after sleeve gastrectomy: a comparative analysis of efficacy and outcomes. Obes Surg..

[19] Wu A, Tian J, Cao L, Gong F, Wu A, Dong G (2018). Single-anastomosis duodeno-ileal bypass with sleeve gastrectomy (SADI-S) as a revisional surgery. Surg Obes Relat Dis..

[20] Zaveri H, Surve A, Cottam D, Ng PC, Enochs P, Billy H, Medlin W, Richards C, Belnap LG, Sharp LS, Bermudez DM, Fairley R, Burns TA, Herrell K, Bull J, Menozzi SE, Student JA (2019). A multi-institutional study on the mid-term outcomes of single anastomosis duodeno-ileal bypass as a surgical revision option after sleeve gastrectomy. Obes Surg..

[21] Balibrea JM, Vilallonga R, Hidalgo M, Ciudin A, Gonzalez O, Caubet E (2017). Mid-term results and responsiveness predictors after two-step single-anastomosis duodeno-ileal bypass with sleeve gastrectomy. Obes Surg..

[22] de la Cruz M, Busing M, Dukovska R, Torres AJ, Reiser M (2020). Short- to medium-term results of single-anastomosis duodeno-ileal bypass compared with one-anastomosis gastric bypass for weight recidivism after laparoscopic sleeve gastrectomy. Surg Obes Relat Dis..

[23] Sanchez-Pernaute A, Rubio MA, Perez N, Marcuello C, Torres A, Perez-Aguirre E (2020). Single-anastomosis duodenoileal bypass as a revisional or second-step operation after sleeve gastrectomy. Surg Obes Relat Dis..

[24] Landreneau JP, Strong AT, Rodriguez JH, Aleassa EM, Aminian A, Brethauer S, Schauer PR, Kroh MD (2018). Conversion of Sleeve Gastrectomy to Roux-en-Y Gastric Bypass. Obes Surg..

[25] Parmar CD, Mahawar KK, Boyle M, Schroeder N, Balupuri S, Small PK (2017). Conversion of sleeve gastrectomy to Roux-en-Y gastric bypass is effective for gastro-oesophageal reflux disease but not for further weight loss. Obes Surg..

[26] Hammer HF (2012). Medical complications of bariatric surgery: focus on malabsorption and dumping syndrome. Dig Dis..

[27] Levinson R, Silverman JB, Catella JG, Rybak I, Jolin H, Isom K (2013). Pharmacotherapy prevention and management of nutritional deficiencies post Roux-en-Y gastric bypass. Obes Surg..

[28] Saltzman E, Karl JP (2013). Nutrient deficiencies after gastric bypass surgery. Annu Rev Nutr..

[29] Brethauer SA, Kothari S, Sudan R, Williams B, English WJ, Brengman M, Kurian M, Hutter M, Stegemann L, Kallies K, Nguyen NT, Ponce J, Morton JM (2014). Systematic review on reoperative bariatric surgery: American Society for Metabolic and Bariatric Surgery Revision Task Force. Surg Obes Relat Dis..

[30] Kuzminov A, Palmer AJ, Wilkinson S, Khatsiev B, Venn AJ (2016). Re-operations after secondary bariatric surgery: a systematic review. Obes Surg..

[31] Zhang L, Tan WH, Chang R, Eagon JC (2015). Perioperative risk and complications of revisional bariatric surgery compared to primary Roux-en-Y gastric bypass. Surg Endosc..

[32] Himpens J, Dapri G, Cadière GB (2006). A prospective randomized study between laparoscopic gastric banding and laparoscopic isolated sleeve gastrectomy: results after 1 and 3 years. Obesity Surgery..

[33] Mandeville Y, Van Looveren R, Vancoillie PJ, Verbeke X, Vandendriessche K, Vuylsteke P (2017). moderating the enthusiasm of sleeve gastrectomy: up to fifty percent of reflux symptoms after ten years in a consecutive series of one hundred laparoscopic sleeve gastrectomies. Obes Surg..

[34] Boru CE, Greco F, Giustacchini P, Raffaelli M, Silecchia G (2018). Short-term outcomes of sleeve gastrectomy conversion to R-Y gastric bypass: multi-center retrospective study. Langenbecks Arch Surg..

[35] Patti MG, Schlottmann F (2018). Gastroesophageal reflux after sleeve gastrectomy. JAMA Surg..

[36] Tack J, Deloose E (2014). Complications of bariatric surgery: dumping syndrome, reflux and vitamin deficiencies. Best Pract Res Clin Gastroenterol..

